# Successful endovascular coil embolization of large pseudoaneurysm of ductus arteriosus diverticulum

**DOI:** 10.1186/s42155-019-0053-5

**Published:** 2019-03-30

**Authors:** David Buechner, Veronica Lippuner, Kristine DeMaio, F. Daniel Donovan, Philip R. Weber, G. Phillip Schoettle

**Affiliations:** 1 Memphis Radiological PC, 7695 Poplar Pike, Germantown, TN 38138 USA; 2 Department of Radiology, University of Tennessee Health Science Center, Memphis, TN USA; 3 Thoracic and Cardiovascular Surgery, Methodist Le Bonheur Healthcare, Memphis, TN USA

**Keywords:** TEVAR, Endovascular coil embolization, Thoracic aneurysm, Ductus arteriosus pseudoaneurysm, Thoracic aortic repair

## Abstract

**Background:**

Pseudoaneurysm of the ductus arteriosus diverticulum, although rare in adults, may have catastrophic consequences if left untreated due to erosion and rupture of the pseudoaneurysm into adjacent thoracic structures. Although thoracic endovascular aortic repair (TEVAR) is the standard treatment method for aneurysm closure of the ductus arteriosus diverticulum, it was not possible in our patient secondary to marked aortoiliac access vessel tortuosity, significant vascular calcific burden, and an abdominal aortic aneurysm. We describe the first reported case of endovascular coil embolization being successfully used as the definite repair of a ductus arteriosus diverticulum pseudoaneurysm.

**Case presentation:**

An 85-year-old man with history of severe coronary arterial disease presented with an enlarging pseudoaneurysm of a ductus arteriosus diverticulum. The diverticulum and thoracic aortic junction demonstrated the typical obtuse angles and wide neck, differentiating it from otherwise similar-appearing diagnostic considerations. Repair was attempted with conventional aortic stent graft but the patient’s infrarenal abdominal aortic aneurysm and his heavily calcified, tortuous iliac vessels could not accommodate the 24Fr introducer sheath necessary for stent graft placement. Therefore, endovascular coil embolization was successfully completed through a 4Fr directional catheter. The patient tolerated the procedure well and was discharged from the hospital in good condition on post-embolization day six.

**Conclusions:**

Endovascular coil embolization is an alternative treatment for ductus arteriosus diverticulum pseudoaneurysm closure in cases where the standard TEVAR method is unsuccessful. Instead of the wide entry point at the aorta we used the junction of the diverticulum and pseudoaneurysm as the “neck” for satisfactory and stable coil placement. Endovascular coil embolization alone may be a viable definitive therapy for occlusion of the ductus pseudoaneurysm component of the diverticulum in cases where complex anatomy or extensive vascular disease makes stent graft repair impractical if not impossible.

## Introduction

Pseudoaneurysm of the ductus arteriosus diverticulum (DAD) is a rare finding in adults. Catastrophic consequences can occur as a result of erosion and rupture into adjacent thoracic structures such as the esophagus, the pericardium, and nearby bronchi (Lund et al., 1991). Additional symptoms can result from compression of these local thoracic structures producing cough, hoarseness, dyspnea, chest pain, neck pain, and dysphagia (Lund et al., 1991; Addams-Williams et al., 2005). Given the critical location and potential for significant complications, it is recommended that, in adults, aneurysms that are either greater than 3 cm in diameter, demonstrate progressive enlargement, or are symptomatic undergo treatment (Mitchell et al., 1983).

In this report, we describe a case of a large DAD pseudoaneurysm that could not be treated with standard aortic stent graft secondary to marked aortoiliac tortuosity, ectasia, abdominal aortic aneurysm, and large vascular calcific burden. This DAD pseudoaneurysm was successfully treated with endovascular coil embolization. There is only one recent report of coil embolization performed in a ruptured ductus arteriosus aneurysm; however, in that case embolization was used as a bridge to definitive endograft and surgical correction (Soeda et al., 2018). Endovascular coil embolization of a DAD pseudoaneurysm without additional thoracic endovascular aortic repair (TEVAR) or open surgical procedure has not been previously reported.

## Case report

**Fig. 1:**
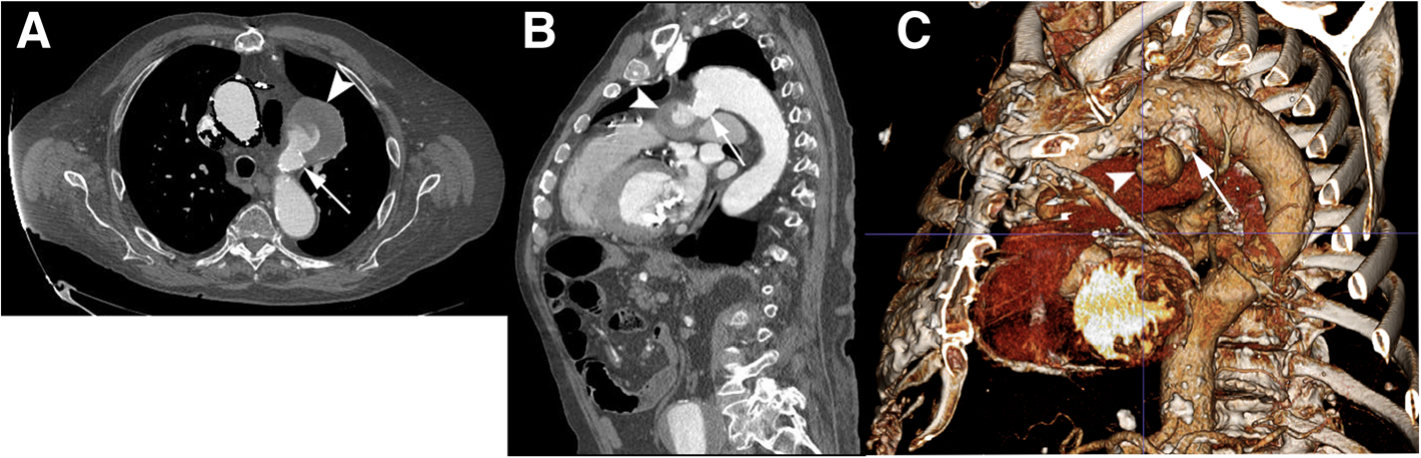
Contrast-enhanced CTA of the thoracic aorta showing the ductus arteriosus diverticulum (DAD) (arrow) and pseudoaneurysm (arrowhead) **a** Axial image demonstrates junction of DAD and pseudoaneurysm **b** Two-dimensional reformatted sagittal plane image delineates the relationship between the aorta, the DAD, and the pseudoaneurysm. **c** Three-dimensional reconstruction of thoracic aorta for visualization and surgical planning

**Fig. 2:**
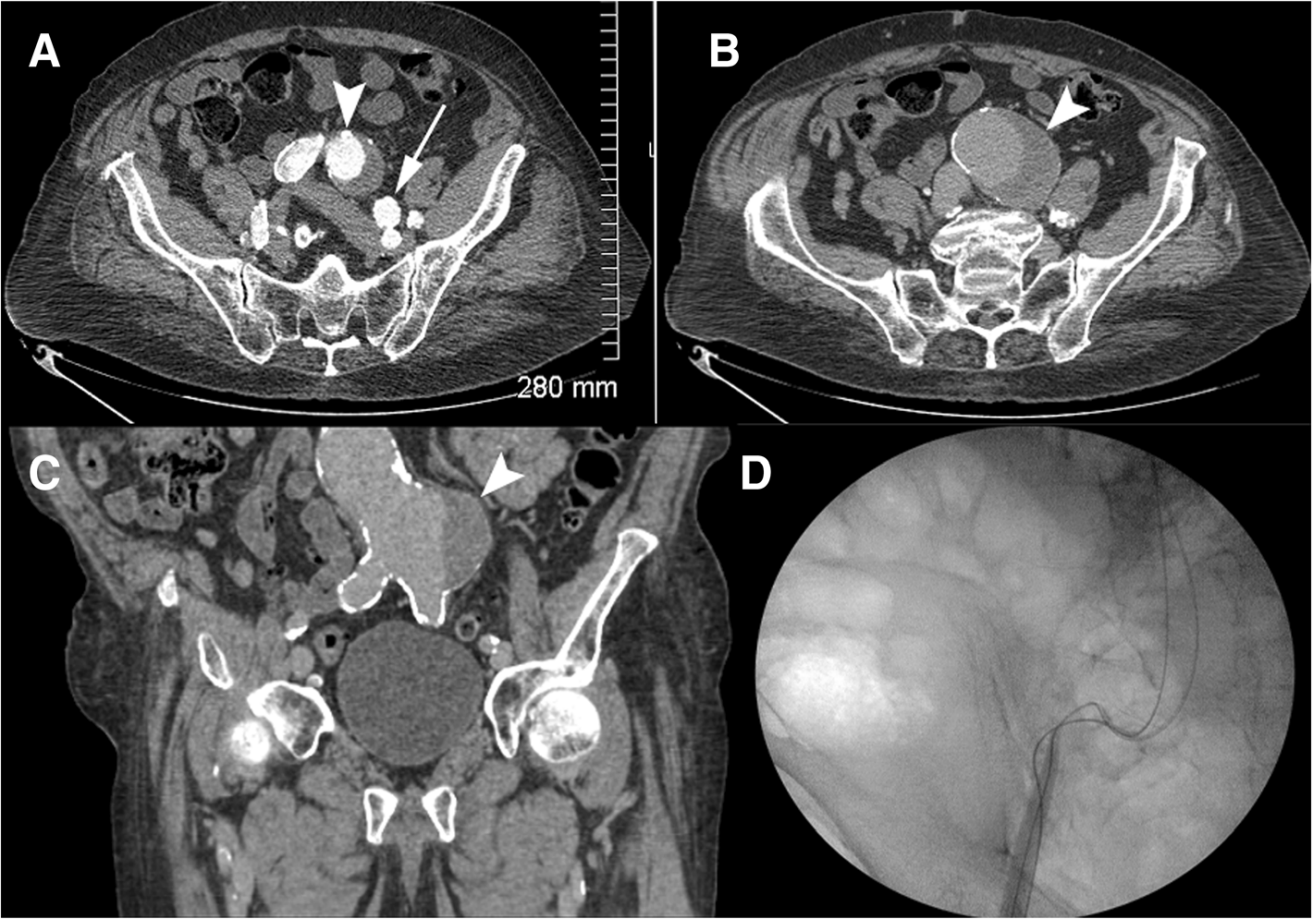
Contrast-enhanced CTA images demonstrate **a.** tortuosity of the iliac vessels (common iliac, arrowhead; external iliac, arrow) **b.** abdominal aortic aneurysm (arrowhead). **c**. Two-dimensional reconstructed sagittal image highlights the infrarenal aortic aneurysm (arrowhead) and iliac and aortic vascular calcifications. **d** Intraoperative fluoroscopic image of the pelvis during endograft TEVAR attempt demonstrates marked aorto-iliac tortuosity

An 85-year-old male patient with known history of severe coronary arterial disease presented for planned repair of his enlarging ductus arteriosus pseudoaneurysm. A previous computed tomography (CT) of the thorax (GE Brightspeed 16 slice Multi Detector CT (MDCT)) from December 2010 demonstrated the pseudoaneurysm which measured 3.6 cm × 3.3 cm in diameter. A follow-up CT angiogram of the chest (GE Lightspeed VCT 64 slice MDCT) in February 2016 demonstrated interval enlargement to a diameter of 6.1 cm × 5.5 cm (Fig. 1). This included the peripheral thrombosed aspect of the pseudoaneurysm as well as the flowing opacified central portion. The flowing central aspect of the pseudoaneurysm measured 2.3 cm. The diverticulum component had a wide neck (1.6 cm) and created obtuse angles with the thoracic aorta (Fig. 1). This diverticular aneurysm was oriented towards the left pulmonary artery, demonstrating that this was indeed a ductus arteriosus pseudoaneurysm. CT angiography (CTA) of the abdomen and pelvis performed concurrently showed severe iliofemoral tortuosity and advanced atherosclerotic calcific burden (Fig. 2). A 5.1 cm infrarenal abdominal aortic aneurysm (AAA) that was directed towards the origin of the left common iliac artery was also demonstrated (Fig. 2b, c).

Endovascular stent graft repair was attempted. The preoperative CTA of the chest suggested that the proximal landing zone of the stent graft would quite likely include the origin of the left subclavian artery. Therefore, a left common carotid to subclavian arterial bypass with interposition ProPaten Graft (Gore, Flagstaff, AZ) was performed to avoid the probable left subclavian artery occlusion.

After the left carotid to left subclavian arterial graft was successfully placed, the right common femoral artery was exposed through a surgical cutdown and the vessel was accessed in a retrograde fashion. This access was to be used for insertion of a 24 French (Fr) introducer sheath used for the introduction of the Medtronic Talent thoracic aortic stent graft system (Minneapolis, MN, USA). Percutaneous access of the left common femoral artery was also obtained for the placement of a 5 Fr flush catheter for the purposes of angiographic control. However, given the previously described advanced common and external iliac tortuosity and severe calcific nature of these vessels combined with the AAA, the 24 Fr introducer sheath could not be successfully navigated distally into the abdominal aorta (Fig. 2d).

**Fig. 3:**
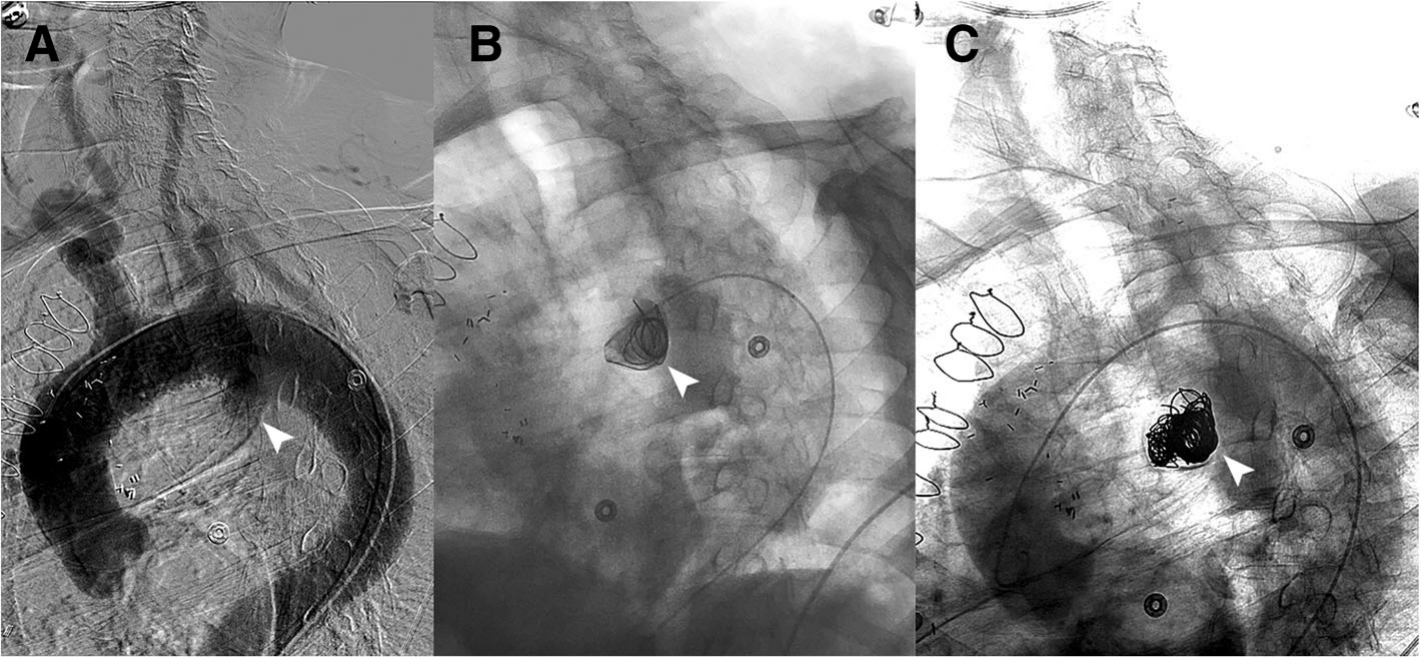
**a** Arch aortogram pre-embolization showing the DAD (arrowhead). **b** Embolization – initial coil placement.**c** Arch aortogram post-embolization demonstrating well-seated coils in the aneurysm sac and no further aneurysm flow

Endovascular coil embolization was performed two days following attempted TEVAR placement. Using right common femoral artery access, a 5 Fr flush catheter (Omni Flush, AngioDynamics, Queensbury, NY) was placed and thoracic angiography was performed. The DAD with its inferior extension of the large pseudoaneurysm component was well visualized (Fig. 3a). A 4 Fr directional catheter (MeritMedical, South Jordan, Utah) was then introduced into the pseudoaneurysm. Given the wide-neck characteristics of the DAD relative to the thoracic aorta, the junction of the diverticulum and the pseudoaneurysm was used as the “neck” and platform for stable endovascular coil placement. A series of eleven Azur 35 detachable coils (Terumo, Somerset, NJ) were placed in the following order: one 20 mm/50 cm framing coil (Fig. 3b), two 20 mm/39 cm coils, two 16 mm/32 cm coils, two 13 mm/24 cm, one 12 mm/30 cm, two 10 mm/19 cm and one 8 mm/13 cm. The loop of the initial framing coil slightly protruded into the diverticulum forming a “bridge” across the junction of the diverticulum and pseudoaneurysm. Follow-up thoracic angiography demonstrated no further flow into the pseudoaneurysm of the DAD (Fig. 3c).

The patient had no immediate complications from the coil embolization. He was taken to the operating room later that night for repair of a right groin hematoma. Ultimately, the patient recovered well and was discharged from the hospital on post-embolization day six. The patient did not return for a recommended three-month-follow-up CTA of the chest. He did, however, present to our facility over a year after the coil embolization, at which point his only concern was a non-healing foot ulcer. He died twenty-one months after the embolization from an acute myocardial infarction with severe underlying coronary artery disease.

## Discussion/conclusions

DAD of the thoracic aorta is a common anatomic embryologic remnant of the ductus arteriosus (Lund et al., 1991). In adults, the reported prevalence of a ductus diverticulum using either catheter directed thoracic angiography or CT angiography is 9–26% (Morse et al., 1988; Hyung Ann et al., 2016). A histopathologic analysis suggested an incidence of up to 44% in adults (Vogler et al., 2007). The primary differential diagnosis of a ductus diverticulum is that of a traumatic thoracic pseudoaneurysm (Hyung Ann et al., 2016). CTA criteria that favor a diverticulum include smaller diameter, obtuse angles with smooth margins, and absence of additional traumatic findings such as aortic dissection flap or hemomediastinum (Hyung Ann et al., 2016).

A pseudoaneurysm of the ductus diverticulum is rare with approximately 150 reported cases, with 74% occurring spontaneously and 26% following surgical closure of a patent ductus arteriosus (Lund et al., 1991). Disease states that are associated with a ductus diverticulum pseudoaneurysm include hypertension and Behçet’s disease as well as connective tissue disorders including Marfan and Ehlers-Danlos syndromes (Müller et al., 1986; Chang et al., 1987).

In this case, high-resolution CTA with volume rendering was used to demonstrate the vascular nature of the mass as well as to differentiate between a traumatic aneurysm versus a true DAD pseudoaneurysm as discussed above. Importantly, CTA is very useful in surgical and endovascular therapeutic planning. Surgical/endovascular access site choice and planning of sheath/stent graft introduction is essential as in all other cases of thoracic aortic aneurysm.

Although TEVAR has become the preferred option for thoracic aortic aneurysm treatment, this procedure requires suitable vascular parameters that consider arterial access route, vascular calcification, vessel tortuosity, stenosis, and overall diameter. In our case, the severely calcific and tortuous iliofemoral route combined with the abdominal aortic aneurysm simply did not allow us to place an aortic stent graft device despite a multitude of endovascular maneuvers.

Coil embolization was performed with satisfactory acute occlusion of the ductus pseudoaneurysm component of the diverticulum. Importantly, here we used the junction of the DAD and the ductus pseudoaneurysm as the platform and “neck” that allowed for stable endovascular coil placement given the “wide neck” and obtuse angles of the junction of the diverticulum and the thoracic aorta. Unfortunately, our patient was lost to follow-up and died twenty-one months after embolization from an acute myocardial infarction. Long-term follow-up CT imaging would be helpful in determining the therapeutic effectiveness of endovascular coil embolization as a potential alternate treatment for occlusion of the ductus pseudoaneurysm component of the diverticulum in cases where complex anatomy or extensive vascular disease makes stent graft repair impractical if not impossible.

## Abbreviations

AAA: abdominal aortic aneurysm
CT: computed tomography
CTA: computed tomography angiography
DAD: ductus arteriosus diverticulum
MDCT: Multi Detector computed tomography
TEVAR: thoracic endovascular aortic repair
